# Neuroimaging Assessment of Cerebrovascular Reactivity in Concussion: Current Concepts, Methodological Considerations, and Review of the Literature

**DOI:** 10.3389/fneur.2016.00061

**Published:** 2016-04-29

**Authors:** Michael J. Ellis, Lawrence N. Ryner, Olivia Sobczyk, Jorn Fierstra, David J. Mikulis, Joseph A. Fisher, James Duffin, W. Alan C. Mutch

**Affiliations:** ^1^Department of Surgery, University of Manitoba, Winnipeg, MB, Canada; ^2^Department of Pediatrics and Child Health, University of Manitoba, Winnipeg, MB, Canada; ^3^Section of Neurosurgery, University of Manitoba, Winnipeg, MB, Canada; ^4^Pan Am Concussion Program, University of Manitoba, Winnipeg, MB, Canada; ^5^Childrens Hospital Research Institute of Manitoba, University of Manitoba, Winnipeg, MB, Canada; ^6^Canada North Concussion Network, University of Manitoba, Winnipeg, MB, Canada; ^7^University of Manitoba, Winnipeg, MB, Canada; ^8^Department of Radiology, University of Manitoba, Winnipeg, MB, Canada; ^9^Health Sciences Centre, University of Manitoba, Winnipeg, MB, Canada; ^10^Institute of Medical Sciences, University of Toronto, Toronto, ON, Canada; ^11^Department of Neurosurgery, University Hospital Zurich, Zurich, Switzerland; ^12^Department of Medical Imaging, University of Toronto, Toronto, ON, Canada; ^13^University of Toronto, Toronto, ON, Canada; ^14^University Health Network Cerebrovascular Reactivity Research Group, Toronto, ON, Canada; ^15^Department of Anesthesia, University of Toronto, Toronto, ON, Canada; ^16^Department of Physiology, University of Toronto, Toronto, ON, Canada; ^17^Department of Anesthesia and Perioperative Medicine, University of Manitoba, Winnipeg, MB, Canada

**Keywords:** concussion, cerebrovascular reactivity, magnetic resonance imaging, blood oxygen level-dependent imaging, carbon dioxide

## Abstract

Concussion is a form of traumatic brain injury (TBI) that presents with a wide spectrum of subjective symptoms and few objective clinical findings. Emerging research suggests that one of the processes that may contribute to concussion pathophysiology is dysregulation of cerebral blood flow (CBF) leading to a mismatch between CBF delivery and the metabolic needs of the injured brain. Cerebrovascular reactivity (CVR) is defined as the change in CBF in response to a measured vasoactive stimulus. Several magnetic resonance imaging (MRI) techniques can be used as a surrogate measure of CBF in clinical and laboratory studies. In order to provide an accurate assessment of CVR, these sequences must be combined with a reliable, reproducible vasoactive stimulus that can manipulate CBF. Although CVR imaging currently plays a crucial role in the diagnosis and management of many cerebrovascular diseases, only recently have studies begun to apply this assessment tool in patients with concussion. In order to evaluate the quality, reliability, and relevance of CVR studies in concussion, it is important that clinicians and researchers have a strong foundational understanding of the role of CBF regulation in health, concussion, and more severe forms of TBI, and an awareness of the advantages and limitations of currently available CVR measurement techniques. Accordingly, in this review, we (1) discuss the role of CVR in TBI and concussion, (2) examine methodological considerations for MRI-based measurement of CVR, and (3) provide an overview of published CVR studies in concussion patients.

## Background

Traumatic brain injury (TBI) continues to be one of the leading causes of death and disability among young people worldwide. The pathophysiology of TBI is characterized by a primary injury that results in structural injury to the brain, followed by a secondary injury that is caused by a combination of inflammation, edema, ischemia, cellular necrosis, and apoptosis (1). Some of these mechanisms are readily detectable on clinical neuroimaging studies in mild, moderate, and severe TBI patients; with the presence of certain features highly correlated with patient outcome (2, 3). Although mTBI and concussion are terms that are often used interchangeably to describe patients with similar clinical presentations, concussion is thought to represent a form of mTBI that typically occurs in the absence of structural brain injury (4). Consequently, clinical neuroimaging studies are normal in the vast majority of patients (5), and the clinical manifestations of this condition are proposed to be mediated by neuronal dysfunction and cerebrovascular dysregulation (6, 7).

Among the most important factors responsible for maintaining brain function during health and injury is the process of matching cerebral blood flow (CBF) to the metabolic needs of the brain. In the setting of TBI, periods of mismatched CBF can result in secondary brain injury that can impact patient outcomes (8–11). Cerebrovascular reactivity (CVR), defined as the change in CBF in response to a vasoactive stimulus, is a means to interrogate the integrity of the effector arm of the demand–supply control of CBF. To measure CVR among patients with cerebrovascular disease including stroke, TBI, and concussion, research studies have combined the use of several advanced non-invasive neuroimaging techniques with several types of vasoactive stimuli. While the neuroimaging techniques used to assess CBF in these studies are highly reliable and reproducible, the vasoactive stimuli used to manipulate CBF are not (12). Several studies have examined CVR in patients with moderate and severe TBI (11, 13), however, only recently have studies examining CVR in patients with concussion become available (14–17). It is therefore important for clinicians and researchers interested in the role of CBF regulation in the pathophysiology of concussion to be able to evaluate the quality, reliability, and implications of studies performed in this population.

Accordingly, the objectives of the present review were threefold: (1) to discuss the role of cerebrovascular physiology in the health and the injured brain including the concussed brain, (2) to review the role and limitations of MRI-based tools and vasoactive stimuli used to measure CVR, and (3) to provide an overview of published CVR studies in concussion patients.

## Cerebral Blood Flow Regulation, Cerebrovascular Reactivity, and Traumatic Brain Injury

### Cerebral Blood Flow Regulation and Cerebrovascular Reactivity

**Figure 1:**
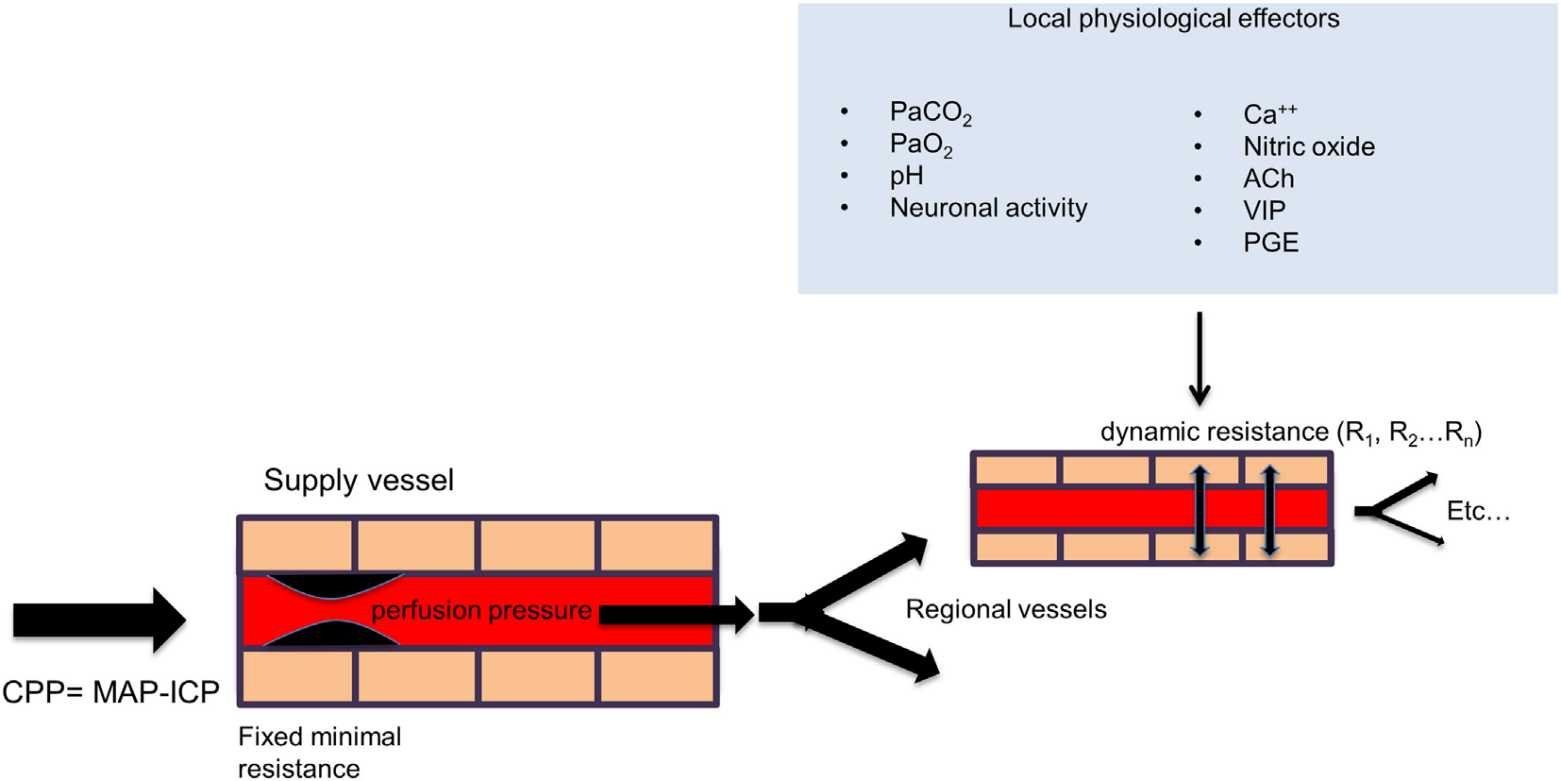
**General schema of cerebral perfusion**. Intracranial vessels are perfused in parallel in a fractal branching pattern. The net flow in each region is dynamically determined by the net flow resistance of each branch. Under normal conditions, the inflow from major extracranial vessels is not limiting. The flow to each vascular region is controlled by its local factors as shown in the figure. The net effect of regional vascular resistances determines the total cerebral blood flow. However, with a strong global vasodilatory stimulus, the drop in resistance in the collective downstream branches can be reduced to the point where the blood flow in the larger supply vessels is limiting (“fixed minimal resistance” in supply vessel in the figure). Abbreviation: CPP, cerebral perfusion pressure; MAP, mean arterial pressure; ICP, intracranial pressure; PaCO_2_, arterial partial pressure of carbon dioxide; PaO_2_, arterial partial pressure of oxygen; Ca^++^, calcium ions; ACh, acetylcholine; VIP, vasoactive intestinal polypeptide; PGE, prostaglandins.

Although the brain accounts for only 2% of total body weight, it receives 15–20% of the cardiac output in humans. In order to maintain the health of this essential organ, the cerebral vasculature must maintain CBF within precise limits under a wide spectrum of states during rest, activity, and disease. Control of CBF is achieved primarily by altering the flow resistance within the cerebral blood vessels at the arteriolar level. In a more global sense, CBF is dependent on the cerebral perfusion pressure (CPP), which is influenced by the mean arterial blood pressure (MAP), the intracranial pressure (ICP), and the resistance of the major arteries. However, on a regional scale, in order to meet the increased energy demands of the brain during activation or disease, the cerebral vasculature must respond appropriately to more localized physiological stimuli, which include the direct action of signaling messengers such as calcium ions, nitric oxide, acetylcholine, vasoactive intestinal polypeptide (VIP), calcitonin gene-related peptide (CGRP), substance P, prostaglandins, and other arachadonic acid metabolites released by astrocytes and neurons as well as local factors such as intraluminal hydrostatic pressure, pH, and partial pressure of arterial carbon dioxide (PaCO_2_) and oxygen (PaO_2_) (18) (see Figure 1). Laboratory evidence also suggests that capillary pericytes respond to similar physiological stimuli and play an important role in CBF regulation during neuronal activation and ischemia at the micro-regional level (19). Failure to appropriately match CBF with surges in the metabolic demands of the brain can lead to temporary or permanent alterations in neurological functioning. This strong dependence is accomplished by a process known as neurovascular coupling, whereby increases in neuronal activity, and consequently, the cerebral metabolic rate of oxygen (CMRO_2_) is tightly related to corresponding increases in regional CBF. Modulation of CBF during neuronal activity requires the coordinated efforts of the neurovascular unit, which includes neurons, astrocytes, pericytes, microglia, and the vascular endothelial and smooth muscle cells (20), and typically results in an “overshoot” in blood flow with respect to O_2_ demand, thereby delivering more oxygen than is consumed by the active assembly of neurons. It has therefore been postulated that this *functional hyperemia* is required for the delivery of other substrates such as glucose, or the removal of metabolic waste or heat. Acute or chronic insufficiency in CBF delivery can exist along a continuum resulting in a wide spectrum of disease conditions including transient ischemic attack (TIA), acute ischemic stroke, and vascular dementia (21). One of the ways to assess the functional integrity of this complex regulatory system is to measure the brain’s cerebrovascular response to a vasoactive stress or stimulus. A vasoactive stimulus is a stimulus that can be applied to the brain and produces a quantifiable and reliable effect on the cerebral vasculature, usually stimulating vasodilation.

**Figure 2:**
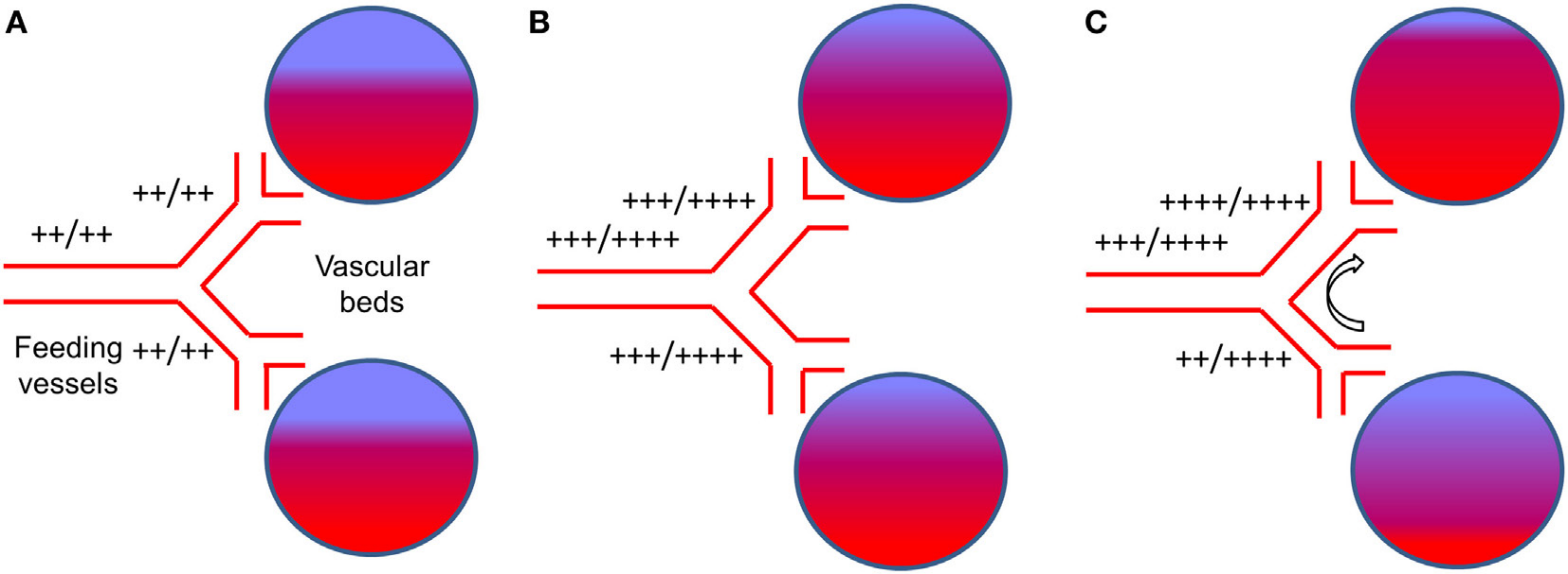
**The effect of a global vasodilatory stimulus on regional blood flow with normal vasculature and with impaired regional vascular response**. **(A)** The normal state at normocapnia. The extent of red color in the vascular beds represents actual blood flow and blue color represents potential blood flow. “++/++” beside vessels represents normal blood flow at rest (++) compared to the flow demand (++). This would be the case for normal vasculature and for vasculature that has branches with reduced vasodilatory capacity. **(B)** With normal vasculature, hypercapnia stimulates increase blood demand by the vascular beds. The vasodilatory demand of the vascular beds combined exceed that of the main feeding vessel (23), which is limiting, i.e., their flow (+++) does not meet demand (++++). However, the dilatory response capability of each feeding vessel is symmetrical and so is their flow. **(C)** In the presence of a dysfunctional vessel, a hypercapnic stimulus results in the same demand in the healthy and dysfunctional vessel (i.e., ++++). There is a strong vasodilation in the healthy (upper) branch and a weaker vasodilation in the dysfunctional (lower) branch. The inflow from the main vessel is still limiting (i.e., +++/++++). The direct competition for flow between the vascular beds results in an increased proportion of the flow through the normal vessel (++++) at the expense of the dysfunctional vessel [flow reduced from +++ in **(B)**, to ++]. This is referred to as vascular ‘steal’.

In general, the effect of any vasodilatory stimulus on any vascular bed in the brain is dependent on (1) the magnitude of the vasodilatory stimulus; (2) the difference in vasodilatory reserve of the interrogated vascular bed and that of the beds with which it is perfused in parallel; and (3) the flow reserve of the feeding vessel to both beds (22) (Figure 2A). Therefore, with intact vasodilatory reserves, the application of a global vasodilatory stimulus like hypercapnia, will produce a balanced reduction in vascular tones and a symmetrical increase in CBF in all beds (Figure 2B). However, if the vasodilatory reserve of a particular vascular bed is compromised, the application of a vasodilatory stimulus will result in an asymmetrical vasodilatory response (Figure 2C). When there is adequate inflow reserve, the changes in flow to each vascular bed follows its change in resistance. When the total flow demand of the beds exceeds the inflow capability, a condition to which the brain vasculature is uniquely susceptible (23), there is a redistribution of blood flow in favor of the vascular bed with better vasodilatory reserve. In severe cases where vasodilatory reserve is diminished or abolished in a vascular bed, the flow becomes completely dependent on the perfusion pressure and on the relative changes in resistance of the vascular beds with intact vasodilatory reserve that are perfused in parallel. Therefore, even in the setting of normal global mean CBF, regional impairments in the control of vascular resistance can lead to regional hypo- and hyperperfusion (see Figure 2C). This mechanism can apply to vessels that range in scale from capillaries to cerebral arteries and even extracranial arteries (24). Such cerebrovascular dysregulation may be unmasked by applying a global vasodilatory stimulus to the cerebral vasculature and observing changes in regional CBF.

While these concepts are easy to understand, they are somewhat difficult to apply in practice. Indeed, CVR varies considerably even among healthy subjects (25) and within a healthy subject over time (26). That CVR normally varies considerably between brain regions and between white matter and gray matter makes accurate assessment and interpretation of CVR studies challenging (27–30). Within these regions, sustained hypoperfusion or the inability to adequately respond to metabolic demand surges (as detected by CVR) can lead to local brain dysfunction (31) and structural degeneration (32). Furthermore, impairments in CVR have been associated with numerous cerebrovascular diseases such as intra- and extracranial steno-occlusive disease (33–37), arteriovenous malformations (38), cerebral proliferative angiopathy (39), and cerebral vasospasm following aneurysmal subarachnoid hemorrhage (40) and are strongly predictive of the development of ischemic stroke (41–43). Given the enormous increases in energy requirements associated with TBI, the adequacy of the cerebrovascular response to match CBF supply to the metabolic demands of the injured brain is likely an important consideration impacting clinical deficits and injury recovery.

### Cerebrovascular Disturbances in TBI

The pathophysiology of TBI is characterized by primary and secondary brain injury. Primary brain injury refers to the direct biomechanical disruption of brain tissue that occurs at the time of initial impact and which can range from focal injuries such as parenchymal contusions and subdural hematoma to more global injuries such as diffuse axonal injury (DAI). While these processes account for a significant proportion of the morbidity and mortality associated with moderate and severe TBI, these processes are not thought to play a prominent role in the pathophysiology of concussion (4). On the other hand, secondary brain injury is characterized by a combination of cellular, metabolic, and inflammatory processes that can result in further tissue edema, injury, and associated neurological deterioration (44, 45). One of the processes that play an important role in secondary brain injury is ischemia. Pathological features of ischemic injury are present in more than 90% of autopsy specimens from patients with fatal TBI (46, 47). Experimental evidence suggests that CBF can fall to 50% of baseline levels in patients with severe TBI (48) and can remain persistently low for weeks to years after injury (49). That this decrease in CBF delivery occurs in the setting of increased metabolic need suggests that the pathophysiology of TBI may have a neurovascular component. On a smaller scale, imbalances in neuronal activity cellular ion transport, increased excitatory neurotransmitter release, and impaired glucose metabolism coupled with insufficient CBF delivery are proposed to account for the “neurometabolic energy crisis” that characterizes acute concussion (6, 7). In addition to concussion and more severe forms of TBI that can occur in sports- and non-sports-related activities, blast-related TBI is a unique form of TBI that presents with similar symptoms and is also characterized by diffuse primary and secondary brain injury processes (50). In particular, animal model evidence suggests that blast-related TBI is associated with time-dependent alterations in many serum proteins responsible for brain cellular metabolic and cerebrovascular processes (51).

Neuroimaging studies in patients with moderate and severe TBI have provided important insights into secondary brain injury and suggest that alterations in CBF and CVR have an important impact on patient outcomes. Bouma et al. (8) found significant correlations between low CBF and clinical motor scores and functional outcomes following severe TBI using Xe-CT. One-third of patients exceeded the ischemic stroke CBF threshold of <18 ml/100 g/min within 6 h of injury, which was associated with higher mortality rates and worse outcomes among survivors. Using positron emission tomography (PET) measurements, Coles et al. (9) found that ischemic brain volumes were significantly higher in severe TBI than controls and correlated with poor 6-month patient outcomes. Wintermark et al. (10) using perfusion-CT found that low regional CBF following TBI was associated with worse 3-month outcomes. Finally, Adelson et al. (11), using Xe-CT showed that mean CBF <20 ml/100 g/min and CVR <2%/mm Hg PaCO_2_ within 2 days of injury were associated with unfavorable outcomes. More recently, Maugans et al. (52) demonstrated impaired mean resting CBF in the acute phase of pediatric sports-related concussion (SRC) that persisted at 1 month despite normalization of neurocognitive testing scores. Bartnik-Olson et al. (53) observed reductions in CBF and cerebral blood volume in the thalamus of pediatric SRC patients compared to normal controls. Meier et al. (54) detected regional abnormalities in resting CBF among collegiate SRC patients during acute injury that were normalized 1 month later. In this latter study, regional CBF within two regions of interest correlated with scores on a neuropsychiatric symptom inventory. Finally, Wang et al. (55) detected regional reductions in CBF within 24 h of injury in collegiate athletes that persisted at day 8 post-injury despite normalization of clinical and neurocognitive testing scores (for summary, see Table 1).

**Table 1:** **Overview of studies examining resting cerebral blood flow in concussion**.

Reference	Study population	Methodology	Clinical concussion measures	Neuroimaging sequence	Results
(52)	12 SRC patients (age 11–15 years) imaged <72 h, 14 days, and 30 days or more post-injury and 12 control subjects	Longitudinal	ImPACT	ASL and DTI MRI	Alterations in mean resting CBF (predominantly reduced CBF) in the acute phase of SRC that persisted at 1 month despite normalization of neurocognitive testing scores
(53)	15 PCS patients (age 8–17 years) imaged 3–12 months post-injury and 15 control subjects	Cross-sectional	Self-reported symptoms	Perfusion-weighted and DTI MRI, MRI-spectroscopy	PCS group demonstrated reduced CBF and CBV in the bilateral thalami compared to the control group. No other significant differences in other brain regions
(54)	15 SRC patients (mean age = 20.57 years) imaged at 0–3 days, 6–13 days, and 25–44 days post-injury and 27 control subjects	Longitudinal	Hamilton Depression and Anxiety rating scales, ANAM	ASL MRI	Reduced CBF in the right dorsal midinsular cortex (dmIC) and superior temporal sulcus in SRC group during acute phase of injury that were similar to the control group at 1 month post-injury
					Regional blood flow within the dmIC was inversely related to the magnitude of initial psychiatric symptoms
(55)	18 SRC patients (mean age = 17.8 years) imaged within 24 h and 8 days post-injury and 19 control subjects	Longitudinal	Sport Concussion Assessment Tool-3, Standardized Assessment of Concussion, Balance Error Scoring System, ANAM, ImPACT	pCASL MRI	At 24 h post-injury reduced CBF was observed in the SRC group within the right supplementary motor area and pre-supplementary motor areas compared to the control group. At 8 days post-injury reduced CBF was observed in diffuse cortical gray matter and thalamus in the SRC group compared to the control group despite normalization of clinical concussion measures

*TBI, traumatic brain injury; SRC, sports-related concussion; PCS, postconcussion syndrome; ImPACT, immediate post-concussion assessment and cognitive testing; ASL, arterial spin labeling; DTI, diffusion tensor imaging; CBF, cerebral blood flow; ANAM, automated neuropsychological assessment metrics*.

Overall, evidence from CVR studies in severe TBI and preliminary studies examining resting CBF in SRC patients supports the conceptual understanding of concussion as characterized by persistent cerebrovascular dysregulation and provides a rationale for examining the role of CVR in patients with concussion.

## Methodological Considerations in MRI-based Measurement of CVR

Over the past 20 years, advances in neuroimaging technology have permitted clinicians and researchers to accurately assess cerebrovascular physiology using a number of techniques. At present, the gold standard for measuring cerebrovascular parameters such as CBF and CMRO_2_ is PET while single photon emission computerized tomography (SPECT) provides comparable assessment of CBF. As previously mentioned, XeCT has also been used to measure CVR in previous studies. Unfortunately, such approaches employ radiation exposure, intravenous administration of contrast agents, and occasionally general anesthesia and intubation with mechanical ventilation to achieve inspired gas control, making these inappropriate techniques for the longitudinal assessment of patients with concussion, especially children and adolescents. Transcranial Doppler ultrasonography has also been used to measure CVR in normal patients and in various cerebrovascular conditions such as concussion (56). It is readily available, inexpensive, and non-invasive; however its poor spatial resolution for CVR, CBF, and tissue perfusion limit its value.

Because CVR represents the unit change in CBF per unit change in a vasoactive stimulus, it is important that both of these variables are measured accurately and applied consistently across study subjects. Failure to do so can lead to differences in CVR measurements between subjects and subject groups that are due to the methodological limitations and variability in techniques and not the disease processes under investigation. Therefore, in order to provide an accurate, reliable, and reproducible MRI-based measure of CVR in humans and any patient population an assessment tool must combine three essential elements: (1) an MRI sequence that can be applied to the entire brain and provide an accurate assessment of CBF or its surrogate at high spatial resolution; (2) a standardized vasoactive stimulus that can provide a measurable and reproducible effect on the cerebral vasculature; and (3) a standardized method of CVR data analysis that allows comparisons between subject groups, and in individual subjects over time.

### Cerebral Blood Flow Assessment

Among the most common non-invasive MRI sequences that are used to assess CBF are blood oxygen level-dependent (BOLD) and arterial spin labeling (ASL). BOLD-level MRI is a technique that exploits the unique MRI signal characteristics of hemoglobin across different oxygenated states within the brain. Simply put, changes in CBF within a specific brain region are associated with changes in the concentration of hemoglobin that transitions between oxygenated and de-oxygenated states within that region. This transition is associated with a quantifiable change in the ratio between oxyhemoglobin (HbO_2_) and deoxyhemoglobin (HHb). HHb is paramagnetic and degrades the BOLD signal in the vessels and surrounding brain tissue. Under normal circumstances, neuronal activation leads to an increase in CBF where the increased delivery of oxygen often exceeds the oxygen consumption of the surrounding tissue (57) resulting in an increase in the HbO_2_/HHb ratio and a resultant increase in the BOLD signal. Within a limited range of CBF, changes in BOLD signal are proportional to reductions in the concentration of HHb. The relationship between the BOLD signal and CBF under a constant CMRO_2_ is fairly linear for CBF increases up to 50% (58), and since CBF changes induced during CVR studies are within this magnitude, the BOLD signal can be used as a surrogate for CBF measurement. However, emerging evidence suggests that the hemodynamic response to neuronal activity is a product of a relationship between neuronal energy consumption and signaling processes (59–61), CBF, CMRO_2_, CBV, and hemoglobin concentration. Thus, under conditions when brain tissue O_2_ consumption increases beyond normal levels (i.e., during injury), this relationship becomes more tenuous.

In contrast, ASL is a technique that can provide a direct measurement of CBF within brain tissue. To accomplish this, a radiofrequency pulse is applied to label incoming blood water protons to invert proton magnetization. These labeled protons can be measured once the blood reaches the perfusion bed of the target tissue. The balance between labeled intravascular water and free water in the tissue is proportionate to the CBF, which can then be modeled using post-processing algorithms to generate CBF in units of ml/100 g tissue/min (62). This technique can be used to measure global and regional resting CBF but must be interpreted with caution in the setting of atherosclerotic steno-occlusive disease where damaged or blocked vessels can increase transit times, leading to delays in arrival of labeled protons and thereby producing errors in the CBF measurement. In fact, there are no publications showing the efficacy of current ASL techniques for measuring CBF quantitatively in patients with advanced cerebrovascular disease. At present, PET remains the only accurate method for doing so but has lower spatial resolution. Nevertheless, attempts to combine ASL with a vasoactive stimulus to measure CVR have shown efficacy when assessing limited brain regions (i.e., non-whole brain coverage) (35). Future refinements of the method (delay insensitive whole brain ASL) may eventually lead to replacement of BOLD for measurement of CVR in advanced cerebrovascular disease.

Taken together, both BOLD and ASL techniques provide accurate, reliable, and reproducible measures of whole brain and regional CBF and can provide similar measures of CVR if paired with a reliable and reproducible vasoactive stimulus.

### Vasoactive Stimuli

In order to provide an accurate measure of CVR between study groups, it is important that assessment tools ensure that subjects are exposed to the same magnitude and duration of vasoactive stimulus (12). This aim can only be accomplished if the vasoactive stimulus produces a quantifiable and reliable effect on the cerebral vasculature and if the magnitude of the stimulus can be measured at the time of administration. In the case of longitudinal studies, it is paramount that the same magnitude of vasoactive stimulus is applied and measured on repeat examinations. If the clinician or researcher cannot confirm that the same stimulus is applied across subjects and during serial assessments within subjects, it is impossible to conclude whether the differences in CVR are a consequence of changes in the disease process or a function of the variability in the study technique (25, 26).

The main vasoactive stimuli that have been used to manipulate CBF in CVR studies in stroke and TBI populations fall into two categories: (1) injection of an exogenous vasodilatory agent (i.e., acetazolamide); (2) modulation of PaCO_2_ *via* inhaled CO_2_, breath-holding, or prospective end-tidal targeting. Each of these techniques has its own advantages and limitations that must be considered in the setting of the patient population under study.

Acetazolamide is a carbonic anhydrase inhibitor that produces a vasodilatory effect on the cerebral vasculature by inducing an intracellular and extracellular acidosis. Since its first introduction to measure CVR in 1986 (63, 64), it has been combined with several neuroimaging techniques to measure CVR in various cerebrovascular disease populations. While intravenous administration of acetzolamide is safe, its side effect profile and effect on CBF are unpredictable. In one study, 63% of subjects administered low dose acetazolamide developed symptoms such as headaches, nausea, dizziness, weakness or numbness of the extremities, and fatigue lasting 0.5–72 h that occurred more frequently in younger patients and females (65). At higher doses, these side effects can be more severe requiring treatment, which in many cases results in prompt termination of the study (66, 67). While these symptoms may be tolerated in normal control subjects and those with asymptomatic cerebrovascular disease, patients with acute SRC and PCS typically have many of these symptoms at rest making prolonged exacerbation of these symptoms undesirable. The need for intravenous access is another disadvantage of this technique, making it less appropriate for use in children and adolescents. However, the most significant disadvantage of this technique for use in CVR studies is the unpredictable pharmacokinetics of acetazolamide administration and its resultant effects on CBF. Serum concentrations following intravenous administration have been found to vary widely between individuals preventing a reliable stimulus–response relationship to be established (12). Even at supramaximal doses, acetazolamide does not produce a maximal CBF response in many normal subjects (66, 67) and can continue to be modulated by the effects of respiratory rate and blood pressure during neuroimaging acquisition. Although some studies have demonstrated that CVR measurements using acetazolamide are comparable to studies using inspired CO_2_ and breath-holding techniques (68–70), they are not reliably so, and there are other techniques that provide a better tolerated and more reliable vasoactive stimulus.

PaCO_2_ is the most potent stimulus for cerebral vasculature vasodilation whereby each millimeter of mercury unit increase is associated by a 2–15% increase in CBF (71, 72). Increases in P_ET_CO_2_ levels of 5–10 mmHg are commonly experienced among children and adults during sleep and physical activities and are well tolerated during studies targeting these levels (73). While administering a fixed “dose” of inspired CO_2_ makes intuitive sense, the PaCO_2_ is not simply a function of the inspired PCO_2_. Rather, it is also a function of the ventilatory response, which is a function of the individual CO_2_ sensitivity that varies between subjects, as will the PetCO_2_ in response to fixed inspired PCO_2_. Since the subject’s ventilatory response cannot be restrained or predicted, neither can the PaCO_2_ (74). Furthermore, the PaCO_2_ (the actual stimulus) is not reliably known as it is loosely related to PetCO_2_ (what is measured), thus reducing the signal-to-noise ratio (SNR) for CVR measurement. Hyperventilation during a hypercapnic challenge can also lead to increases in end-tidal O_2_ (P_ET_O_2_) and PaO_2_ (up to 15 mmHg) (75), which can result in an increase in BOLD signal that is independent of the PaCO_2_ changes leading to falsely elevated CVR values (76). Furthermore, the BP response to CO_2_ can also vary considerably among normal control subjects and can act as an additional confounding variable for accurate CVR measurement (29). Altogether, there are numerous individual differences that influence CVR measurement; factors that are difficult to account for unless PetCO_2_ PetO_2_, HR, BP, and ventilatory rate are rigorously and frequently measured during image acquisition.

For those studies that use block design CO_2_ breathing protocols, the rates of change of PaCO_2_ and PaO_2_, are unpredictable, other than knowing that no plateau is likely to be achieved within the duration of a protocol. Because CVR represents the change in CBF per unit change in CO_2_, some studies have used PetCO_2_ as a surrogate for PaCO_2_. However, the differences between PetCO_2_ and PaCO_2_ depend on a number of factors including age, exercise, body composition, and other features of respiratory physiology. Even under conditions of accurate and frequent PetCO_2_ and PetO_2_ measurement, CO_2_ inhalation generates an imprecise vasoactive stimulus and therefore an imprecise measure of CVR.

Another technique that has been long used to generate a hypercapnic stimulus in CVR studies is breath holding. The primary advantage of this technique is it requires no breathing circuits or equipment to administer CO_2_. A simplifying assumption is that breath-holding results in a linear rise in PaCO_2_ with time. In this way, the magnitude of the vasoactive stimulus is conceptually quantified according to the duration of breath-holding. However, the rise of PaCO_2_ with time is not linear; the tolerance for duration of breath-holding varies widely between people (68); the PaO_2_ drops precipitously with time; and the PetCO_2_ at the end of breath-holding is not necessarily equal to the PaCO_2_. Consequently, there is no consensus on how breath-holding data should be analyzed as there is no stimulus information (no knowledge of PaCO_2_ levels) and selection of an appropriate regressor to regress against the BOLD data for generation of global CVR maps is problematic. Finally, as breath-holding relies on repeated sustained Valsalva maneuvers, it is undesirable for use in patients with acute SRC and PCS who, in our clinical experience, frequently report an exacerbation of their concussion symptoms with this type of exertion.

**Figure 3:**
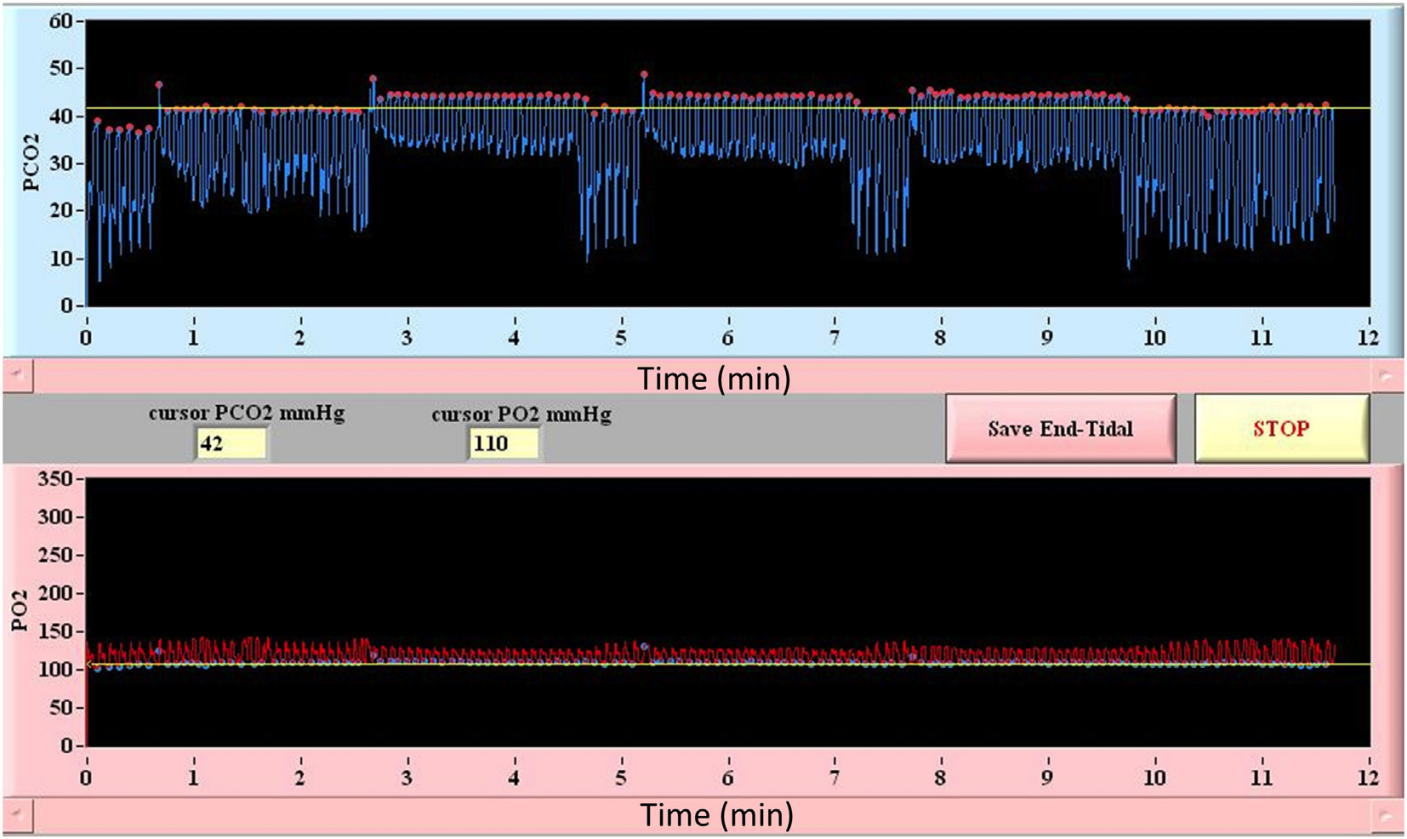
**Block design breathing protocol using model-based prospective end-tidal ETCO_2_ and ETO_2_ targeting**. Triple hypercapnic stimulus during controlled iso-oxic conditions is illustrated. Breath-by-breath confirmation of ETCO_2_ and ETO_2_ allows for accurate measurement and interpretation of CVR assessments.

Among currently available techniques, only automated computer controlled gas blenders have proved capable of delivering a precise, highly controlled, and measurable hypercapnic stimulus for CVR studies (75, 77). These techniques are capable of precise manipulation of PetCO_2_ under iso-oxic conditions. In this way, the CBF changes that result from changes in PaCO_2_ are not adversely effected by the unaccounted changes in PaO_2_. They also enable various patterns of gas challenges with transitions such as a step change, ramp, or multi-block design that can be tailored to the patient population and permit statistically rigorous assessment of CVR (24, 25, 71, 78). The prospective targeting method includes end-inspiratory rebreathing assuring the equality of the PetCO_2_ and PaCO_2_ (79, 80) and providing the same vasoactive stimulus in comparative and longitudinal studies; an important requirement that can not be met by other techniques previously described here (26) (an example of prospective end-tidal targeting is provided in Figure 3). Prospective targeting is safe (all inspired gases contain O_2_) and well tolerated and requires only a minimal amount of cooperation on the part of study subjects. Using this technique, children as young as 10 years of age with cerebrovascular disease (81) and as young as 13 years of age with concussion (17) have undergone successful CVR imaging. Most importantly, the rigorous control of end-tidal gases permitted by this technique translates into precise voxel tracking during BOLD image acquisition and highly reliable and reproducible between (22) and within-subject measures of CVR (26, 73). This level of performance however comes with some disadvantages compared to other techniques including the need for specialized equipment including the breathing circuits, gas blender, and customized gases as well as the need for on-site personnel that have experience in respiratory physiology and mechanics and are trained to operate the equipment.

When compared, both inhaled CO_2_ and breath-holding have been found to provide an inferior assessment of absolute CVR compared to prospective targeting (82). Although associated with an increased investment in cost and personnel, prospective targeting is the only available technique capable of providing an accurate, reliable, and reproducible vasoactive stimulus and thus optimal assessment of CVR.

### Data Analysis

The primary goal of CVR data analysis is to assess whether global or regional differences in CVR observed among a patient or a group of patients are significantly different compared to those observed among healthy control subjects and that the characteristics of the disease under investigation are responsible for this difference. Because of the wide variability in structural brain characteristics and CVR values that can occur in healthy subjects, it is important that CVR data analysis techniques provide a standardized method of subject comparison that control for these subtleties. Accomplishing this aim requires the generation of a sizeable normal control atlas that can account for the immaterial variables that can potentially contribute to changes in CVR including age, sex, hemoglobin concentration, physical fitness level, time of day, hormone levels, state of mind, and technical factors related to the vasoactive stimulus and MRI equipment (25). Importantly, as it relates to concussion, it is also necessary that the control atlas include patients with a history of previous but recovered concussion so that observed changes in CVR can be reliably attributed to the most recent and symptomatic concussion and not just the presence of any lifetime concussion. At present, there are several CVR data analysis techniques that can be used to facilitate these comparisons.

A detailed description of the application of such a technique has been described by Sobczyk et al. (25). A reference atlas is constructed using Analysis of Functional NeuroImages (AFNI) software to co-register each of the individual T1-weighted anatomical images into a common Montreal Neurological Institute 152 space (MNI152) using a calculated transformation subsequently applied to the BOLD data. In order to generate a CVR reference atlas, each healthy subject’s anatomical and BOLD data (whether normal control or patient) are normalized into the same MNI152 space and time aligned with PetCO_2_ allowing CVR to be calculated for each voxel. The mean (M) ± SD of CVR for each voxel location is calculated. In a test scan, the CVR of the corresponding voxel is scored according to its *z* score [*z* = (*M* − CVR)/SD] by comparison to the reference atlas. This normalizes the voxel CVR score for anatomical location and provides a probability score of its being within the normal range. The magnitude of the *z* score (larger numbers implying low probabilities of being normal) is color-coded and superimposed onto the subject’s anatomic scan providing a comprehensive view of the distribution of normal and abnormal responses in the scan. This CVR scoring technique therefore takes into account the voxel’s anatomical location as well as the normal variability. It would be particularly important for the study of concussion, where the injury may be diffuse or isolated in particular anatomical regions.

**Figure 4:**
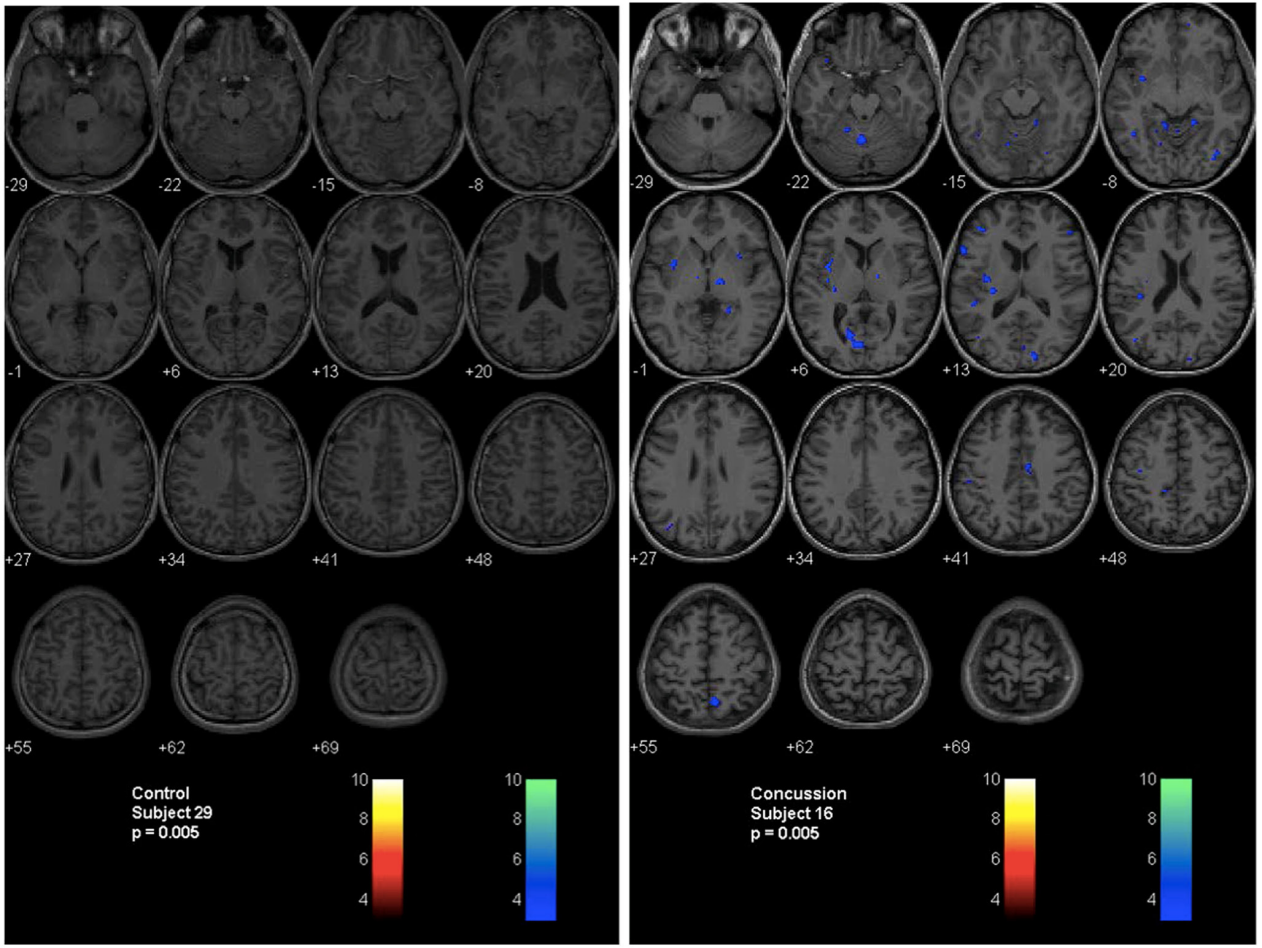
**Second-level analysis maps and postconcussion symptom scale scores for healthy control subject and adolescent postconcussion syndrome patient**. Second level individual comparisons examined at the *p* = 0.005 level demonstrate no evidence of abnormal voxels in the healthy control subject compared to the atlas of normal controls (left panel). Quantitative patient-specific alterations in cerebrovascular responsiveness are demonstrated in the adolescent postconcussion syndrome patient (right panel).

**Figure 5:**
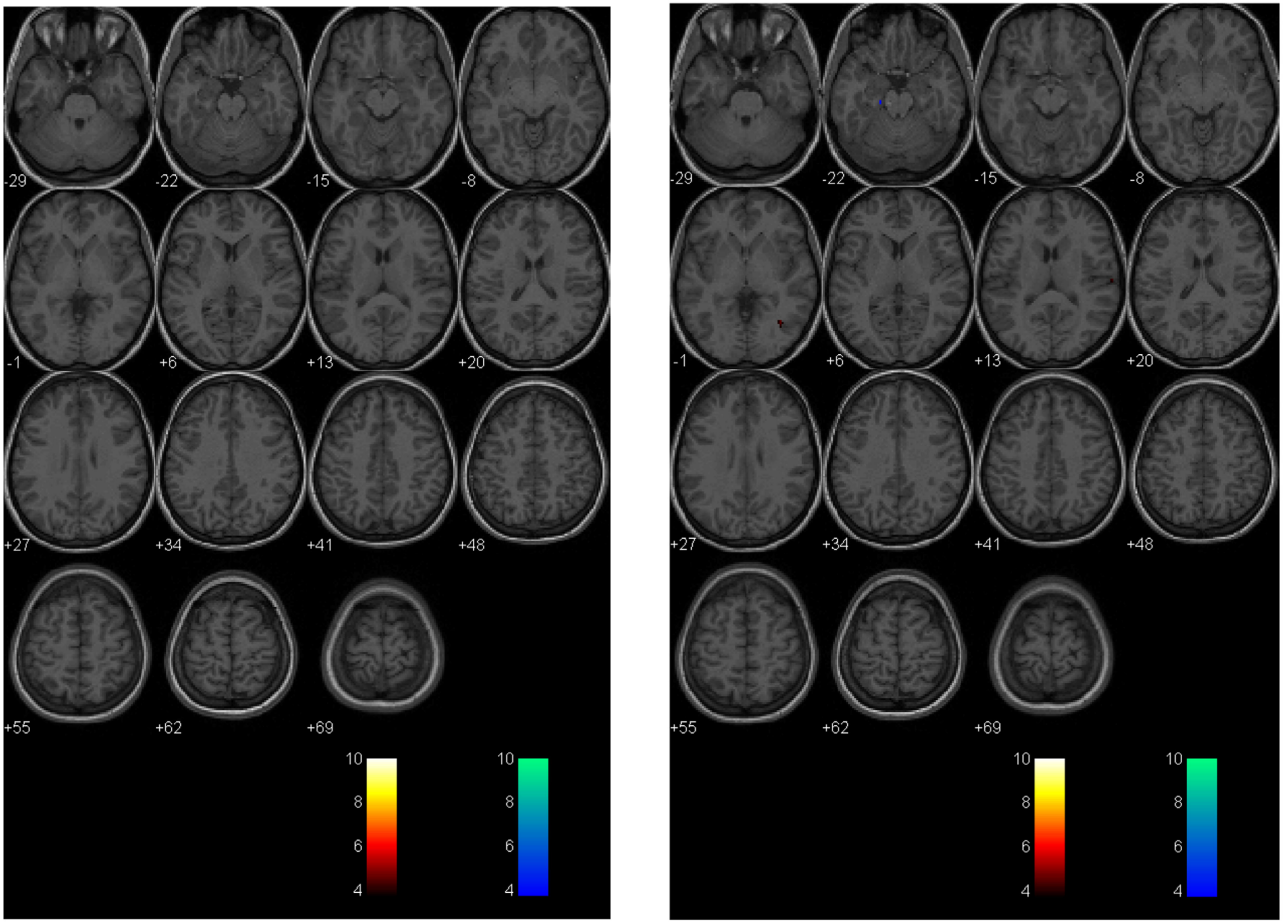
**Longitudinal assessment of healthy control subject**. Second-level analysis in a healthy adolescent imaged 18 months apart and compared to a normal atlas reveals stable CVR assessment. The *p*-value is 0.005 as in Figure 4.

**Figure 6:**
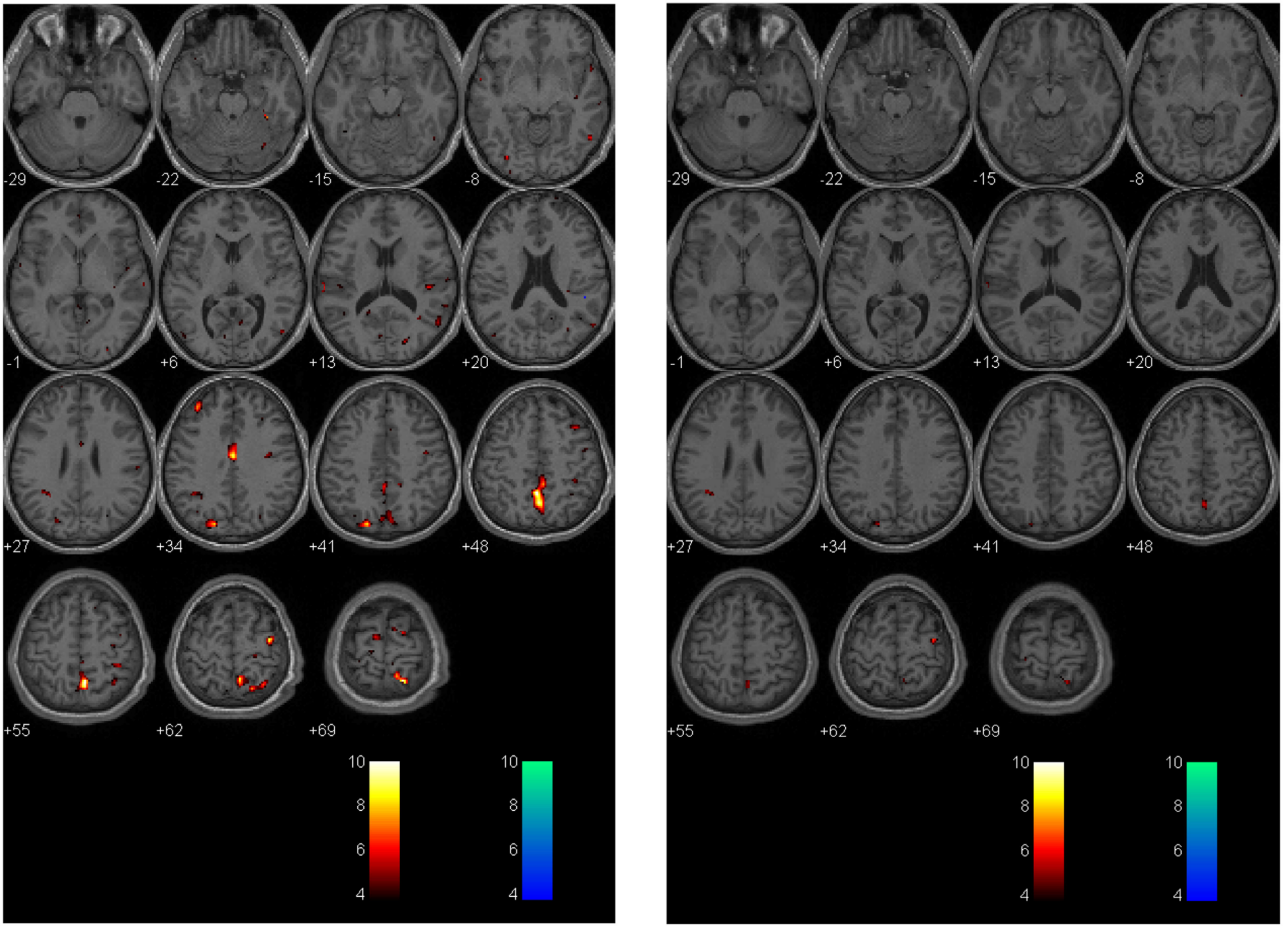
**Longitudinal assessment of adolescent postconcussion syndrome patient**. Symptomatic adolescent PCS patient imaged following abnormal formal neuropsychological testing and symptom-limiting threshold on graded aerobic treadmill testing 3 months post-injury [left panel; image reproduced with permission from Journal of Neurosurgery [Mutch et al. (17)]. Patient re-imaged 5 months later following treatment with sub-maximal exercise prescription resulting in symptom, neuropsychological, and physiological recovery (right panel). The *p*-value is 0.005 as in Figures 4 and 5.

A second technique described by Mutch et al. (14, 17, 71) uses a similar methodology. In this technique, anatomical and BOLD data from the normal control subjects are post-processed using SPM, normalized into MNI152 space and time-aligned with the PetCO_2_ output to generate a normal CVR atlas. To assess voxel-by-voxel differences in the CVR response between an individual subject (control or patient), second-level analyses can be completed under conditions that examine the number of voxels that are greater than or less than those observed in the control atlas with different levels of statistical significance. In this way, voxel-by-voxel differences can be color coded and overlaid onto the anatomical maps to provide a qualitative assessment of regional CVR abnormalities (Figure 4). More importantly, quantitative biomarkers for voxels greater than, less than, or the sum of these (total abnormal voxel counts) can be generated and compared to other objective measures of the condition under investigation. Similar to the *z*-scores described above, these quantitative biomarkers can be used to longitudinally track CVR abnormalities over time in individual subjects or patients (Figures 5 and 6).

In addition to evaluating global and regional voxel-wise differences in CVR between normal control subjects and patients, additional modifications to these techniques can be used to measure the *rate* of CBF increase to a controlled vasoactive stimulus. Using the prospective targeting approach for CO_2_ delivery, subjects undergo an abrupt (within two breaths) step change in PetCO_2_ during BOLD imaging acquisition (24). Analysis can then proceed by one of two methods. First, both the steady state CVR and the rate of change of the BOLD response (expressed as a time constant of change for an exponential function or tau) can be calculated on a voxel-by-voxel basis. These measurements can be color-coded similar to the raw CVR data and overlaid onto the subject’s anatomical maps to generate tau and steady state CVR maps that can be analyzed in a fashion analogous to the *z*-map technique (24). Second, transfer function analysis can also be applied, which provides even greater information about the dynamic BOLD response to hypercapnia including gain, coherence, and phase (78). Preliminary studies suggest that the onset and time course of the CBF response to a vasodilatory stimulus is prolonged in patients with other cerebrovascular diseases such as steno-occlusive disease and Alzheimer’s disease (24, 78). To date, there have been no studies that have examined the dynamic BOLD response to a vasodilatory stimulus in TBI or concussion.

## Overview of CVR Studies in Concussion

The preceding discussion of cerebrovascular pathophysiology in TBI, and CVR measurement techniques, provides a perspective from which to evaluate the quality and applicability of previous (and future) studies that examine CVR in patients with concussion (Table 2).

**Table 2:** **Methodological framework for evaluation of cerebrovascular reactivity measurement in concussion**.

Questions	
(1)	What is the patient population being investigated (e.g., acute concussion, sports-related concussion, postconcussion syndrome, mTBI)?
(2)	What is the study methodology (e.g., cross-sectional and longitudinal)?
(3)	What clinical measures are used to assess concussion patients (e.g., symptom inventory, neurocognitive tests)?
(4)	Are the control group subjects appropriate for comparison to the patient population?
(5)	What neuroimaging sequences are used to assess cerebral blood flow (e.g., BOLD MRI, and ASL)?
(6)	What vasoactive stimulus is used to manipulate cerebral blood flow?
(7)	Is the magnitude of the vasoactive stimulus measured and reported?
(8)	Is the magnitude of the vasoactive stimulus equal between groups and within subjects?
(9)	What are the results of CVR measurement (e.g., group differences and individual differences)?
(10)	Does the CVR assessment technique yield any quantitative biomarkers?
(11)	What is the relationship between the CVR results and other neuroimaging findings?

*mTBI, mild traumatic brain injury; BOLD, blood oxygen-dependent level; ASL, arterial spin labeling; CVR, cerebrovascular reactivity*.

We searched for published studies that met the following criteria: (1) included a study population of one or more patients with concussion or mTBI and (2) utilized MRI-based techniques to measure CVR or cerebrovascular responsiveness. Overall, four studies met the inclusion criteria and were analyzed (results summarized in Table 3).

**Table 3:** **Overview of studies examining cerebrovascular reactivity in traumatic brain injury and concussion**.

Reference	Study population	Methodology	Clinical concussion measures	Control group	Neuroimaging sequence	Vasoactive stimulus	Results	Patient-specific quantitative CVR biomarker	Other findings
(13)	30 severe TBI patients (age 1 month–8 years) imaged at admission and up to 9 days post-injury	Longitudinal	Glascow Coma Scale, Glascow Outcome Score	None	Xenon-CT	Mechanical ventilation	Baseline ETCO_2_ = not reported Delta CO_2_ = average 8.4 Torr; range 5–11 Torr, CVR = CVR <2%/Torr PaCO_2_ associated with poor outcome	(1) Whole brain CVR	Mean CBF ≤20 ml/100 mg/min associated with poor patient outcome at any time during study
(11)	95 severe TBI patients (age 0.1–18.4 years) imaged at admission and up to 9 days post-injury. 38 patients underwent CVR imaging	Longitudinal	Glasgow Coma Scale, Glasgow Outcome Score	None	Xenon-CT	Mechanical ventilation	Baseline ETCO_2_, delta CO_2_ = not reported CVR = CVR <2%/Torr PaCO_2_ associated with unfavorable outcome	(1) Whole brain CVR	Mean CBF on admission associated with patient outcome
			Unfavorable outcomes seen in all patients with CBF ≤20 ml/100 mg/min during post-injury day 0–2					
(14)	12 concussion patients: 8 symptomatic PCS, 4 asymptomatic (age 19–46 years, 11 males, 1 female) imaged 1–12 months post-injury	Cross-sectional study	Postconcussion symptom scale	5 males (age 27–41 years)	BOLD MRI	Prospective end-tidal targeting	Baseline ETCO_2_, delta CO_2_ = no difference between groups CVR = No differences in whole brain CVR. Patient-specific impairments in CVR observed among concussion patients and not healthy controls	(1) Whole brain CVR (2) Whole brain abnormal voxel counts	None
(15)	47-year-old female mild TBI patient imaged at 2 months and 1 year post-injury	Longitudinal study	None	5 males (aged 27–35 years)	BOLD MRI	Breath-holding	Baseline ETCO_2_, delta CO_2_ = not reported CVR = hemispheric asymmetry in CVR observed at initial study. Less asymmetry observed at follow-up study	None	None
(16)	7 SRC patients (age 19–22, 4 males: 3 females) imaged 3–6 days following injury	Cross-sectional study	Rivermead Post-Concussion Symptoms Questionnaire	11 subjects (age 18–23 years, 5 males: 6 females)	BOLD MRI	Inhaled CO_2_	Baseline ETCO_2_, delta CO_2_ = not reported CVR = Higher mean CVR values within regions of interest in the concussion group compared to control group	None	No difference in resting CBF within regions of interest between concussion group and healthy control group using pCASL MRI CVR increased within some default mode networks Increase functional connectivity within hippocampus related to increased CVR
(17)	15 PCS patients (age 15–22 years, 4 males: 11 females) imaged 1–33 months following injury	Cross-sectional study	(1) Postconcussion symptom scale (2) Graded aerobic treadmill testing	17 subjects (12–21 years, 8 male: 9 female)	BOLD MRI	Prospective end-tidal targeting	Baseline ETCO_2_, response, delta CO_2_ = no difference between groups CVR = mean CVR impairments observed between PCS group and healthy control group Patient-specific impairments in CVR observed among individual PCS patients not healthy controls	(1) Whole brain abnormal voxel counts	No difference in mean resting CBF between PCS patients and healthy controls using pCASL MRI Regional impairments in resting CBF among PCS patients compared to healthy controls Patient-specific alterations in regional CBF in PCS patients and not healthy controls

*mTBI, mild traumatic brain injury; SRC, sports-related concussion; PCS, postconcussion syndrome; BOLD, blood oxygen-dependent level; pCASL, pseudo-continuous arterial spin labeling; CVR, cerebrovascular reactivity; ETCO_2_, end-tidal carbon dioxide; MRI, magnetic resonance imaging; CT, computerized tomography*.

Among these, Mutch et al. (14) utilized prospective iso-oxic targeting of PetCO_2_ and BOLD MRI to examine cerebrovascular responsiveness in adult concussion patients and normal control subjects. In this pilot study, they observed no differences in PetCO_2_ changes and whole brain CVR between subject groups. However, they demonstrated quantified patient-specific alterations in CVR that were present in both symptomatic and asymptomatic concussion patients that were absent among healthy control subjects. Chan et al. (15) performed a longitudinal study on a single mTBI patient during the symptomatic and asymptomatic stages of mTBI. Using breath-holding during BOLD MRI, they observed qualitative asymmetries in whole brain CVR on the initial assessment that were less asymmetrical on follow-up assessment. Miltana et al. (16) used inhaled CO_2_ during BOLD MRI to evaluate CVR in a group of acute SRC patients and healthy control subjects. They observed CVR values within certain regions of interest that were higher in the SRC group compared to the control group. Increased functional connectivity within the hippocampus correlated with increased CVR values. No group differences were observed in resting mean CBF measured with pCASL. Finally, Mutch et al. (17) utilized prospective PetCO_2_ and BOLD MRI to examine cerebrovascular responsiveness in adolescent PCS patients and normal controls. With a standardized PetCO_2_, there was a reduced cerebrovascular responsiveness observed in the PCS group. Patient-specific qualitative and quantitative alterations in cerebrovascular responsiveness were observed in all individual PCS patients. Preliminary receiver operator curve analysis revealed an area under the curve of 0.87 (*p* < 0.0001) for voxels manifesting a CVR response greater than the control response and 0.80 (*p* = 0.0003) for those manifesting a CVR response lower than the control response. No differences in global mean CBF measured with pCASL were observed; however, patient-specific alterations in regional resting CBF were observed among PCS patients.

## Comments

Although recent neuroimaging studies have advanced our understanding of concussion and mTBI, a diagnostic assessment tool that provides qualitative and quantitative assessment of brain pathophysiology in this clinical population has remained elusive. Despite the increasing popularity and application of neuroimaging tools such as diffusion tensor imaging, task-based and resting state functional MRI, and MRI-based assessment of resting CBF in the academic literature, none of these techniques are presently capable of providing clinically meaningful information that impacts the management of individual concussion patients (83, 84). However, in its relatively short history, MRI-based CVR imaging has the potential to play an instrumental role in the diagnosis, prognostication, and management of several cerebrovascular disorders. Because TBI and concussion are thought to be associated with alterations in cellular metabolism and CBF that are similar to those observed in stroke, the emerging concept of concussion as a form of cerebrovascular disease is novel and intriguing. Consequently, there is hope that MRI-based CBF and CVR studies will not only uncover the pathophysiological mechanisms governing individual concussion symptoms but may also provide a means to accurately and reliably contribute to the diagnosis, classification, prognostication, and confirmed recovery in individual acute concussion and PCS patients.

As future studies venture into this evolving landscape, it is important for clinicians and researchers to have a thorough appreciation of the role of CBF and CVR in TBI, and have an understanding of the methodological limitations of the neuroimaging assessment tools that are used to investigate these processes. As discussed here, currently available MRI sequences provide an accurate, reliable, and reproducible measure of CBF. Unfortunately, the vasoactive stimuli used to manipulate CBF, and therefore, permit accurate measurement of CVR are not methodologically equal. Readers are reminded that in order to provide accurate, reliable, and reproducible measures of CVR that the magnitude of the vasoactive stimulus must be measurable and equal across subjects, otherwise conclusions regarding the effects of a disease process on cerebrovascular physiology can not be reliably made. This issue is sufficient to account for the paucity of reliable and comparable data regarding the role of cerebrovascular dysfunction in concussion, to date.

For MRI-based CVR assessment to impact the management of individual concussion patients, a number of unique challenges must be overcome. Unlike cerebrovascular diseases such as stroke which have well validated neuroimaging and clinical outcome measures against which CVR findings can be compared, the severity of concussion and its effect on subjective physical, cognitive, and emotional functioning are not easily measured. Many of the symptoms of concussion are non-specific and found in other conditions such as primary headache disorders, whiplash, depression, and endorsed among normal subjects in the absence of TBI (85–90). Although neurocognitive and neuropsychiatric tools can provide an objective measure of some of these processes, there remain a significant proportion of concussion patients in whom these studies are normal despite ongoing symptoms. Not only does this reinforce the need to apply a multi-disciplinary approach to concussion diagnosis and recovery assessment but also suggests that neuroimaging studies that generate potential quantitative biomarkers that rely on an association with scores based on other objective tools such as these must be interpreted with caution and may not be generalizable to broader acute concussion and PCS populations. Most importantly, because patients with concussion may have pre-existing conditions or develop neurological complications or disorders such as depression, migraine headaches, and other neurodegenerative diseases such as chronic traumatic encephalopathy that may also have a cerebrovascular etiology, the potential for using this technology as a diagnostic and medical clearance tool will rely on its ability to reliably distinguish between concussion and these pre-existing and post-injury disorders. Given the enormity of factors that influence CVR in normal healthy subjects and the likelihood that the alterations in CVR that occur in concussion are much more subtle than those that characterize other cerebrovascular disease, at present, the availability of a precise reproducible vasoactive stimulus reduces the SNR optimizing the opportunity to identify the cerebrovascular biomarkers associated with this condition.

In summary, preliminary research suggests that concussion is a heterogeneous condition that presents with patient-specific alterations in cerebrovascular physiology that can be safely and reliably assessed using validated MRI-based CBF and CVR assessment techniques. Future studies are warranted to determine the magnitude and natural history of these alterations following acute concussion, their relationship to other objective concussion assessment tools, and whether these alterations can be ameliorated through the application of targeted rehabilitation strategies.

## Ethics Statement

Images included in this manuscript were obtained from a study approved by the University of Manitoba Biomedical Research Ethics Board. Informed patient and parental consent (for subjects 18 years of age and younger) was obtained for all research subjects (postconcussion patients and normal control subjects). This research did not include any vulnerable populations, persons with disabilities, or endangered animal species.

## Author Contributions

Conception and design of the work (all authors). Drafting the work and revising it critically for important intellectual content (all authors). Final approval of the version to be published (all authors). Agreement to be accountable for all aspects of the work ensuring that questions related to the accuracy or integrity of any part of the work are appropriately investigated and resolved (all authors).

## Conflict of Interest Statement

The authors or their affiliated institutions have not received any payment or services from a third party for any aspect of the submitted work. DM, JF, and JD are senior scientists at Thornhill Research Inc. (TRI), a company affiliated with the University Health Network and University of Toronto that developed the RespirAct, a patented, non-commercial research tool assembled by TRI to enable cerebrovascular reactivity studies.
